# Risk prediction models for venous thromboembolism in lung cancer patients after surgery: a systematic review and meta-analysis

**DOI:** 10.3389/fonc.2026.1767471

**Published:** 2026-05-20

**Authors:** Tenglu Sun, Yuanyuan Chen, Xuli Shang, Haifang Lin, Yongxia Wang, He Wei, Fei Yang

**Affiliations:** 1 School of Medicine, Lishui University, Lishui, Zhejiang, China; 2 Department of Nursing, Lishui Hospital of Wenzhou Medical University, The First Affiliated Hospital of Lishui University, Lishui People's Hospital, Lishui, Zhejiang, China

**Keywords:** lung cancer, meta-analysis, postoperative, risk prediction model, venous thromboembolism

## Abstract

**Background:**

Risk prediction models for venous thromboembolism (VTE) in lung cancer patients undergoing surgery have increased substantially in recent years. However, the methodological quality, predictive performance, and clinical applicability of these models have yet to be systematically assessed.

**Objective:**

This study aimed to systematically evaluate the published literature on the development and validation of postoperative VTE risk prediction models for patients with lung cancer.

**Design:**

A systematic review and meta-analysis of observational studies was conducted.

**Methods:**

A comprehensive search of CNKI, Wanfang, VIP, PubMed, Web of Science, The Cochrane Library, CINAHL, and Embase was conducted from inception to November 22, 2025. The data extracted from the included studies encompassed a range of characteristics, including design elements, predictors, model development strategies, validation approaches, and performance metrics. The Prediction Model Risk of Bias Assessment Tool (PROBAST) was utilized to evaluate the risk of bias and applicability. A meta-analysis of area under the receiver operating characteristic curve (AUC) values from validated models was performed using random-effects methods.

**Results:**

A total of 4,700 records were identified, and after screening, twenty studies involving twenty prediction models were included. The majority of the studies were retrospective and single-center, and all were adjudged to have a high risk of bias according to PROBAST. Logistic regression emerged as the predominant modeling approach, while a limited number of studies adopted machine learning methods, including XGBoost and stacked models. The most frequently utilized predictors were D-dimer and age. The extent of reported model discrimination exhibited significant variability, with AUC values ranging from 0.66 to 0.99. A total of eight models that had undergone validation were deemed eligible for the quantitative synthesis, resulting in a pooled AUC of 0.85 (95% confidence interval [CI]: 0.78–0.93). However, substantial heterogeneity was observed (*I*² = 89.1%).

**Conclusion:**

While several models showed some discriminatory ability, all included studies demonstrated a high risk of bias and limitations in applicability. The extant evidence does not support the routine clinical use of existing postoperative VTE prediction models in lung cancer patients. Future studies should adopt rigorous methodological frameworks, ensure adequate sample sizes, apply standardized predictor handling, and conduct multicenter external validation to improve the reliability and clinical utility of prediction models.

**Systematic review registration:**

https://www.crd.york.ac.uk/prospero/, identifier CRD420251232098.

## 1 Background

Lung cancer remains a leading cause of cancer-related mortality worldwide, accounting for approximately 18% of all cancer deaths and posing a substantial burden on global health systems. Venous thromboembolism (VTE), comprising deep vein thrombosis and pulmonary embolism, constitutes a substantial and potentially fatal postoperative complication in patients with lung cancer (1, 2). Surgical resection, a prevalent treatment modality for lung cancer, has been demonstrated to markedly augment the risk of VTE. Reported postoperative VTE incidence after lung cancer surgery varies widely across studies, likely owing to differences in patient characteristics, screening strategies, thromboprophylaxis practices, and follow-up duration; a recent systematic review and meta-analysis estimated a pooled incidence of 1.82%, whereas nationwide cohort data have shown that VTE remains clinically relevant and occurs most commonly within the first 3 months after surgery (3). The initial postoperative period is of paramount importance, as factors such as immobility, cancer-related hypercoagulability, and surgical trauma synergistically increase the risk of thrombosis (1). VTE has been demonstrated to have a significant impact on both short-term morbidity and mortality rates, as well as long-term survival outcomes in patients diagnosed with lung cancer. The presence of VTE has been identified as an independent predictor of unfavorable long-term survival outcomes in these patients (4). Furthermore, postoperative mortality is often attributable to pulmonary embolism, which frequently originates from deep vein thrombosis (1).

A VTE risk prediction model uses multiple predictors to estimate an individual’s risk of thrombosis. These predictors encompass various elements, including patient demographics, laboratory parameters, and cancer-specific features (5). Due to the fact that VTE manifests after lung cancer surgery in a manner that is frequently accompanied by subtle or absent symptoms, the implementation of reliable prediction tools can assist clinicians in implementing timely thromboprophylaxis and personalized monitoring (6). At present, general risk assessment models such as the Caprini and Khorana scores are frequently used in clinical practice to assess VTE risk in patients with cancer (7, 8). However, their derivation settings differ substantially from the postoperative lung cancer context: the Caprini score was originally designed as a broad perioperative risk assessment tool for surgical patients, whereas the Khorana score was developed for ambulatory patients initiating chemotherapy. Therefore, neither model was specifically calibrated for the combined effects of thoracic surgical trauma, lung cancer-associated hypercoagulability, and perioperative coagulation disturbances in patients undergoing lung cancer surgery. Consequently, the development and validation of lung cancer-specific prediction models has gained increasing attention. Despite the recent proliferation of models in this field, there is a paucity of systematic evaluations regarding the methodological rigor, predictive performance, and clinical applicability of these models. The present systematic review and meta-analysis aims to address this knowledge gap by identifying, evaluating, and synthesizing the available risk prediction models for VTE in lung cancer patients after surgery, with the overarching objective of providing an evidence base to guide clinical decision-making and future research.

## 2 Methods

The study protocol was duly registered on PROSPERO (registration number: CRD420251232098).

### 2.1 Search strategy

To conduct a comprehensive search, we targeted both Chinese and English databases, considering the large population size and language universality. The databases searched included China National Knowledge Infrastructure (CNKI), Wanfang Database, China Science and Technology Journal Database (VIP), PubMed, Web of Science, The Cochrane Library, Cumulative Index to Nursing and Allied Health Literature (CINAHL), and Embase. The search was conducted from database inception to November 22, 2025, using combinations of the following terms: “venous thromboembolism,” “deep vein thrombosis,” “pulmonary embolism,” “lung cancer,” “surgery,” “postoperative,” “risk prediction model,” “risk factor,” “predictor,” “model,” and “risk score.” Additionally, a comprehensive review of the reference lists of the retrieved studies and review articles was conducted to identify additional relevant studies.

The Population, Intervention, Comparison, Outcome, Timeframe, and Study design (PICOTS) framework was used to structure the review, as recommended by the Critical Appraisal and Data Extraction for Systematic Reviews of Prediction Modelling Studies (CHARMS) checklist (9). The system’s functionality encompasses the delineation of the review’s objective, the formulation of its search strategy, and the establishment of its study inclusion and exclusion criteria (10). The following section delineates the fundamental components of our systematic review.

P (Population): This study focuses on patients who have undergone surgical procedures for lung cancer.

I (Intervention model): Published risk prediction models for postoperative VTE in lung cancer patients (predictors ≥ 2).

C (Comparator): No competing models.

O (Outcome): The occurrence of VTE (rather than its subtypes) is of particular interest.

T (Timing): The prediction is based on baseline information collected at hospital admission, including results from clinical scoring scales and laboratory parameters.

S (Setting): The model is designed for the individualized prediction of VTE in post-operative lung cancer patients, thereby facilitating the implementation of preventive measures to avoid adverse events.

### 2.2 Inclusion and exclusion criteria

Studies were included if they involved patients undergoing surgery for lung cancer, used an observational study design, reported a postoperative VTE prediction model, and defined VTE as the outcome of interest.

Studies were excluded if they did not develop a prediction model, examined only VTE subtypes rather than overall VTE, were not published in English or Chinese, or had unavailable full texts despite attempts to contact the authors by email.

### 2.3 Study selection and screening

The screening process for the studies was conducted independently by two authors (Sun Tenglu and Yangfei). Initially, duplicate studies were removed, and the remaining studies were assessed based on their titles and abstracts to determine their eligibility. Following the application of the inclusion and exclusion criteria, the full texts of all relevant studies were reviewed, and the reference lists of these studies were examined to identify any potentially relevant studies. In the event of conflicting opinions regarding the selection of a study, a deliberative process involving three authors (Sun Tenglu, Yangfei, and Chen Yuanyuan) was initiated to achieve a consensus.

### 2.4 Data extraction

The initial screening of the search results was conducted by two independent reviewers. The eligibility of the full-text reports was assessed, and any discrepancies were resolved through discussion or by a third reviewer.

Data extracted from the included studies were grouped into two categories. The first category included basic study characteristics, namely author, publication year, study design, participants, and data source. The second category included model-related information, namely variable selection method, model development method, validation type, performance measures, handling of missing data, processing of continuous variables, final predictors, and model presentation. One reviewer performed the data extraction, and a second reviewer checked the extracted data for accuracy and consistency.

### 2.5 Quality assessment

The risk of bias and applicability of the included studies were assessed using the Prediction Model Risk of Bias Assessment Tool (PROBAST) (11). Two authors (Sun Tenglu and Yangfei) independently evaluated the presence of bias and concerns regarding the applicability of the studies. The PROBAST checklist is a tool that can be utilized for the critical appraisal of studies involved in developing, validating, or updating prediction models for individualized predictions. The instrument under review comprises 20 signaling questions, which are categorized into four domains: participants, predictors, outcome, and analysis. Each signaling question is to be answered with one of the following responses: “yes,” “probably yes,” “no,” “probably no,” or “no information.” In the event that a minimum of one signaling question within a given domain is answered in the negative or with a “probably no,” the domain in question should be regarded as being at high risk of bias. The overall risk of bias can only be adjudged low when all domains are deemed to be low risk.

### 2.6 Data synthesis and statistical analysis

A meta-analysis of the area under the curve (AUC) values from the validated models was conducted using R software (version 4.5.1; R Foundation for Statistical Computing, Vienna, Austria) in RStudio. Quantitative synthesis was restricted to AUC estimates derived from post-development validation rather than apparent development-set performance, according to published methodological guidance for systematic reviews and meta-analyses of prediction model performance (10). When both internal and external validation AUCs were available, the external validation estimate was preferentially used. Standard errors were extracted from reported 95% confidence intervals when available; when confidence intervals were not reported, standard errors were estimated from the AUC value and sample size using the Hanley and McNeil method (12, 13). Because substantial between-study heterogeneity was anticipated in predictors, modeling strategies, validation approaches, and target outcomes, pooled AUC estimates were synthesized using a random-effects model (14). Heterogeneity was assessed using the *I*² statistic and Cochran’s Q test. *I*² values of 25%, 50%, and 75% are indicative of low, moderate, and high heterogeneity, respectively (15). Publication bias and small-study effects were not formally assessed because fewer than ten studies were included in the quantitative synthesis (16).

## 3 Results

### 3.1 Study selection

**Figure 1:**
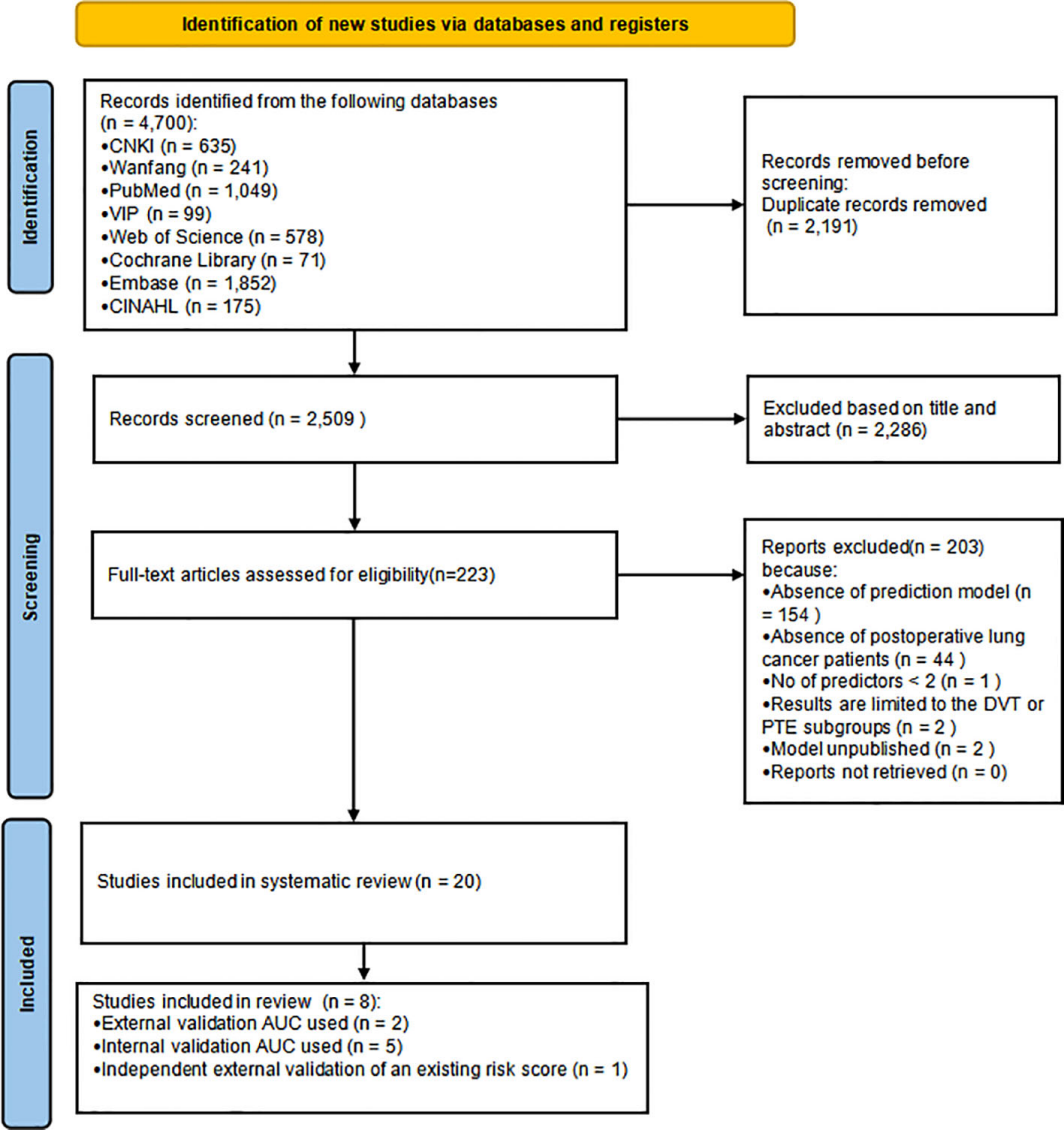
Preferred reporting items for systematic reviews and meta-analyses (PRISMA) flowchart of literature search and selection.

**Figure 1** shows the Preferred Reporting Items for Systematic reviews and Meta-Analyses (PRISMA) 2020 flowchart depicting the comprehensive search process and results.

The initial search yielded a total of 4,700 indexed records. After removing 2,191 duplicate records identified across all databases, 2,509 titles and abstracts were screened for eligibility. After title and abstract screening, 223 articles were retained for full-text assessment. During full-text review, 154 studies were excluded because they did not develop prediction models. Additionally, 44 studies were excluded because their study populations were inconsistent with the target population of the review, one study included fewer than two predictors, two studies limited the outcome to VTE subtypes, and two studies did not present a prediction model. No full-text articles were excluded because the reports could not be retrieved after attempts to contact the authors by email. Ultimately, twenty studies with twenty models were included in this review.

### 3.2 Study characteristics

**Table 1** summarizes the characteristics of the included studies and their participant profiles. A total of 20 studies published between 2016 and 2025 were included in the analysis, with 19 conducted in China and one in the United States. In terms of study design, two studies were prospective single-center studies, 17 were retrospective studies, including one multicenter study, and one was a multicenter cross-sectional study. The study populations mainly comprised patients undergoing surgery for lung cancer, including five studies of non–small cell lung cancer (NSCLC), three studies of elderly patients aged >60 years, one study of synchronous multiple primary lung cancers, and 11 studies involving mixed or unspecified lung cancer subtypes.

**Table 1:** Overview of basic data of the included studies.

Author/year	Country	Participants	Study design	Data source	Scope of surgery	Type of surgery	Outcome indicator observation time	Main outcome	Cases/ sample size
Cai et al. (2023) (21)	China	Patients with pathological stage IA NSCLC undergoing lung resection from 2017–2022	Retrospective study	Thoracic surgery department of a hospital	LOB, Segmentectomy, Wedge Resection	VATS, thoracotomy	Postoperative period until discharge	DVT	40/452
Li Y. et al. (2021) (22)	China	Patients with lung cancer undergoing lung resection from 2015-2018, aged ≥60 years	Retrospective matched case-control study	Thoracic surgery department of a hospital	LOB, Pneumonectomy, Segmentectomy, Wedge Resection	VATS, thoracotomy	Within 30 days postoperatively	PTE	136/680
Lin et al. (2025) (32)	China	Patients with lung cancer undergoing surgery from 2023 to 2024 aged ≥60 years	Retrospective study	Thoracic surgery department of a hospital	–	VATS, thoracotomy	Within 6 months postoperatively	VTE	55/320
Qin et al. (2023) (23)	China	Patients with primary lung cancer undergoing lung resection from 2020-2022	Retrospective study	Thoracic surgery department of a hospital		VATS, thoracotomy	Postoperative period until discharge	VTE	138/502
Tang et al. (2025) (24)	China	Patients with sMPLC from 2017 to 2024 aged ≥ge years	Retrospective study	Thoracic surgery department of a hospital	LOB, Segmentectomy, Wedge Resection, Segmentectomy+wedge	VATS	Within 1 month after discharge	VTE	148/1,984
Chen C. et al. (2025) (33)	China	Patients with NSCLC undergoing radical surgery from 2019 to 2023	Retrospective study	Multicenter study	LOB, Segmentectomy	–	Within 30 days postoperatively	VTE	80/472
Li J. et al. (2024) (34)	China	Patients with lung cancer who underwent surgical treatment between 2019 and 2023, aged ≥60 years	Retrospective study	One hospital	–	–	At 3 months postoperatively	DVT	84/352
Li P. et al. (2024) (25)	China	Patients with NSCLC undergoing radical surgery from 2016 to 2022	Retrospective study	One hospital	Radical surgery	VATS, thoracotomy	Postoperative period	DVT	95/217
Liu H. et al. (2024) (26)	China	Patients with NSCLC undergoing surgery from 2019 to 2023 aged ≥60 years	Retrospective study	Thoracic surgery department of a hospital	–	VATS	During hospitalization	DVT	32/354
Liu Y. et al. (2024) (27)	China	Patients with lung cancer undergoing radical surgery from 2021 to 2023 aged ≥60 years	Retrospective study	One hospital	LOB, Segmentectomy	VATS	Postoperative period	VTE	34/102
Zhang et al. (2022) (28)	China	Patients with lung cancer undergoing surgery from 2017 to 2021	Retrospective study	One hospital	LOB, Pneumonectomy, Segmentectomy, Wedge Resection	VATS	Postoperative period	VTE	59/118
Ding et al. (2023) (35)	China	Patients with stage I–III lung cancer who underwent surgery between 2019 and 2021, aged ≥60 years	Prospective cohort study	One hospital	LOB, Segmentectomy, Wedge Resection	VATS, thoracotomy	Postoperative period until discharge	VTE	63/601
Chen Z. et al. (2025) (17)	China	Patients with lung cancer undergoing surgery from April 2021 to December 2023	Retrospective study	Thoracic surgery department of a hospital	–	–	Perioperative period	VTE	175/1,013
Hao et al. (2025) (18)	China	Patients with NSCLC undergoing surgery from January 2018 to November 2022 aged ≥60 years	Retrospective study	Thoracic surgery department of a hospital	LOB	–	Within 28 days postoperatively	DVT	58/362
Li J. et al. (2023) (36)	China	Patients with lung cancer undergoing surgery from June 2017 to February 2019 aged ≥60 years	Prospective case-control study	One hospital	–	VATS, thoracotomy	Within 30 days postoperatively	DVT	71/445
Ke et al. (2022) (19)	China	Patients with primary lung cancer undergoing surgery from August 2016 to December 2019 aged ≥60 years	Multicenter cross-sectional cohort study	Multicenter study	LOB, Pneumonectomy, Segmentectomy, Wedge Resection	VATS, thoracotomy	Postoperative period until discharge	VTE	87/1,205
Hachey et al. (2016) (20)	the United States	Patients with resectable lung cancer from 2005 to 2013	Retrospective study	Thoracic surgery department of a hospital	Segmentectomy, LOB, Pneumonectomy	VATS, thoracotomy	Within 60 days after surgery	VTE	12/232
Peng et al. (2025) (29)	China	Patients with newly diagnosed lung cancer from August 2023 to August 2024 aged 18–84 years	Retrospective study	One hospital	–	VATS, thoracotomy	Postoperative period, not clearly specified	VTE	31/203
Jin et al. (2025) (30)	China	Patients with lung cancer from June 2018 to June 2019	Retrospective study	Thoracic surgery department of a hospital	LOB, Segmentectomy, Wedge Resection, Other	thoracotomy, thoracoscopic surgery	Postoperative period	DVT	20/125
Yang et al. (2022) (31)	China	Elderly patients with lung cancer from February 2018 to February 2022 aged >60 years	Retrospective study	Thoracic surgery department of a hospital	LOB, Segmentectomy	VATS	Within 30 days postoperatively	DVT	61/183

“-”, not reported; DVT, deep venous thrombosis; VTE, venous thromboembolism; PTE, pulmonary thromboembolism; LOB, lobectomy; VATS, video-assisted thoracoscopic surgery; NSCLC, non–small cell lung cancer; sMPLC, synchronous multiple primary lung cancer.

**Table 2** presents the model information from the included studies. Among the included studies, 16 developed prediction models using multivariable logistic regression, reflecting its dominant role in clinical risk modeling. Chen Z. et al. used the XGBoost algorithm, whereas Hao et al. used a stacked machine learning approach (17, 18). Furthermore, Ke et al. developed a modified Caprini risk assessment model, and Hachey et al. conducted a validation study of the original Caprini model (19, 20). The most frequently utilized predictive factors were D-dimer-related indicators and age, which appeared in 15 and nine models, respectively. Other commonly identified predictors included Caprini-related scores and operation time/surgical duration, each of which was reported in seven models. Preoperative chemotherapy was identified in four models. Reported AUC or C-statistic values ranged from 0.66 to 0.99. A total of 15 models underwent calibration assessment, with calibration plots being the most commonly used method.

**Table 2:** Overview of the information of the included prediction models.

Author/year	Missing data handling	Continuous variable processing method	Variable selection	Model development method	Calibration method	Validation method	Predictive factor	Model performance	Model presentation
Cai et al. (2023) (21)	Complete case analysis	Continuous variable	Multivariate analysis after univariate screening	Multivariable logistic regression model	Calibration plot	Internal validation	Age, Preoperative D-dimer, Intermuscular vein dilatation	A: 0.832 (0.732–0.924) B: 0.791 (0.668–0.930)	Nomogram model
Li Y. et al. (2021) (22)	Multiple Imputation	Continuous variable	Multivariate analysis after univariate screening	Multivariable logistic regression model	Hosmer-Lemeshow test	Internal validation	Age, BMI, Operation time, Preoperative CA15-3, Preoperative CUS	A: 0.793 (0.734–0.853) B: 0.813 (0.737–0.890)	Nomogram model, Formula of risk score
Lin et al. (2025) (32)	Complete case analysis	Primarily categorical (CRP as continuous)	Univariate screening followed by Lasso regression	Multivariable logistic regression model, l1-regularized regression model	Hosmer-Lemeshow test, Calibration plot	Internal validation, External validation	Caprini score, Operation time, Surgical approach, Clinical stage, Preoperative chemotherapy, Preoperative D-dimer, CRP	A: 0.966 (0.948–0.985) B: -	Nomogram model
Qin et al. (2023) (23)	Complete case analysis	Continuous variable	Multivariate analysis after univariate screening	Multivariable logistic regression model	Hosmer-Lemeshow test, Calibration plot	Internal validation	Age, Operation time, FEV1, Postoperative TEG K value, Postoperative TEG R value	A: 0.913 (0.867–0.958) B: 0.955 (0.917–0.993)	Nomogram model, Formula of risk score
Tang et al. (2025) (24)	Complete case analysis	Continuous variable	Univariate screening followed by Lasso regression	Multivariable logistic regression; LASSO used for variable selection	Calibration plot	Internal validation	Age, Smoking history, Coronary artery disease, Cerebrovascular disease, COPD, Atherosclerotic plaques in the extremities, Surgical approach, Intraoperative transfusion, Postoperative Caprini score, Number of primary lesions, Preoperative D-dimer	A: 0.917 (0.894-0.941)	Nomogram model
Chen C. et al. (2025) (33)	–	Continuous variable	Multivariate analysis after univariate screening	Multivariable logistic regression model	Calibration plot	Internal validation, External validation	Age, TNM stage, Operation time, Preoperative D-dimer	A: 0.836 B: 0.864 (0.783-0.945)	Nomogram model
Li J. et al. (2024) (34)	Complete case analysis	Continuous variable	Multivariate analysis after univariate screening	Multivariable logistic regression model	Hosmer-Lemeshow test, Calibration plot	–	Diabetes mellitus, Hyperlipidemia, Preoperative chemotherapy, Postoperative bed duration, FAR, SII, Caprini score	A: 0.825	Nomogram model
Li P. et al. (2024) (25)	Complete case analysis	Categorical variables	Multivariate analysis after univariate screening	Multivariable logistic regression model	Hosmer-Lemeshow test, Calibration plot	Internal validation, External validation	Age, Diabetes mellitus, Pathological type, Clinical stage	A: 0.900 (0.839-0.960) B: 0.903 (0.854-0.951)	Nomogram model
Liu H. et al. (2024) (26)	–	Continuous variable	LASSO regression, univariate screening prior to multivariate analysis	Multivariable logistic regression; LASSO used for variable selection	Calibration plot	Internal validation	D-dimer, CVC, Lower Extremity Varicose Veins	A: 0.912 (0.840-0.983)	Nomogram model
Liu Y. et al. (2024) (27)	Complete case analysis	Categorical variables	Multivariate analysis after univariate screening	Multivariable logistic regression model	Calibration plot	–	Surgical Approach, Operation time, Preoperative D-dimer, Postoperative bed duration	A: 0.898	Nomogram model
Zhang et al. (2022) (28)	–	Continuous variable	Multivariate analysis after univariate screening	Multivariable logistic regression model	–	–	Preoperative Carcinoembryonic Antigen, Operation time, D-dimer at 8 Hours Postoperatively	A: 0.902 (0.848-0.955)	Formula of risk score
Ding et al. (2023) (35)	Complete case analysis	Continuous variable	Multivariate analysis after univariate screening	Multivariable logistic regression model	–	–	Modified Caprini risk assessment model; extended model adds Hb and D-dimer	A: 0.822 (0.760-0.855)	–
Chen Z. et al. (2025) (17)	Bayesian ridge imputation for numerical variables; missing category for categorical variables	Continuous variable	Univariate screening followed by Lasso regression	Lasso-based feature selection followed by machine learning model development	Calibration plot	Internal validation	Age, mean corpuscular volume, mean corpuscular hemoglobin, fibrinogen, D-dimer, and albumin	A: 0.99 B: 0.66 (0.57-0.75)	Online prediction tool
Hao et al. (2025) (18)	Complete case analysis	Continuous variable	Multivariate analysis after univariate screening	Multivariable logistic regression-based feature selection and machine learning model development	Calibration plot; Brier score	Internal validation	Age, platelets, D-dimer, albumin, smoking history, and EGFR exon 21 mutation	A: 0.984 B: 0.983 (0.93-1.00)	–
Li J. et al. (2023) (36)	Complete case analysis	Continuous variable	Multivariate analysis after univariate screening	Multivariable logistic regression model	–	Internal validation	Thoracoscopic resection (1 day postoperatively): R value (Coagulation Reaction Time), K value (Clot Formation Rate), α angle, MA,FIB, D-dimer, MDA, CD4^+^/CD8^+^ Thoracotomy resection (3 days postoperatively): R value, K value, α angle, D-dimer, MDA, SOD	VATS model: A: 0.934 Thoracotomy model: A: 0.918	Formula of risk score
Ke et al. (2022) (19)	Complete case analysis	Continuous variable	Multivariate analysis after univariate screening	Modified Caprini risk assessment model	–	–	Standard Caprini items, Modified Surgical duration, Elevated D-dimer	A: 0.759 (0.710–0.808)	Formula of risk score
Hachey et al. (2016) (20)	Complete case analysis	Continuous variable	–	Validation of the Caprini risk assessment model	Hosmer-Lemeshow test	Independent validation of an existing risk score	Caprini risk score	B: 0.78 (0.62-0.94)	Caprini risk score
Peng et al. (2025) (29)	Complete case analysis	Categorical variables	Multivariate analysis after univariate screening	Multivariable logistic regression model	Calibration plot	–	Age ≥60 years old, complicated with diabetes mellitus, clinical stage III preoperative chemotherapy, prolonged hospital stay, elevated D-dimer level	A: 0.874 (0.821–0.927)	Nomogram model
Jin et al. (2025) (30)	–	Continuous variable	Multivariate analysis after univariate screening	Multivariable logistic regression model	–	–	Modified Caprini score; D-dimer	A: 0.800 (0.689–0.892)	–
Yang et al. (2022) (31)	Complete case analysis	Continuous variable	Multivariate analysis after univariate screening	Multivariable logistic regression model	Calibration plot	Internal validation	Preoperative chemotherapy, Surgical method, Postoperative bed rest time, Combined with diabetes, Preoperative D-dimer	A: 0.86 (0.81–0.93)	Nomogram model

A, development cohort; B, validation cohort; “-”, not reported; BMI, body mass index; CA15-3, cancer antigen 15-3; CUS, compression ultrasonography; CRP, c-reactive protein; TEG, thromboelastography; FEV1, forced expiratory volume in 1 second; COPD, chronic obstructive pulmonary disease; FAR, fibrinogen to albumin ratio; SII, systemic immune-inflammation index; CVC, central venous catheter; EGFR, epidermal growth factor receptor; MA, maximum amplitude; FIB, fibrinogen; MDA, malondialdehyde; SOD, superoxide dismutase; VTE, venous thromboembolism; NSCLC, non–small cell lung cancer; sMPLC, synchronous multiple primary lung cancers; TNM, tumor–node–metastasis; CEA, carcinoembryonic antigen; LOB, lobectomy; VATS, video-assisted thoracoscopic surgery; DVT, deep vein thrombosis; PTE, pulmonary thromboembolism; MCV, mean corpuscular volume; MCH, mean corpuscular hemoglobin; AUC, area under the receiver operating characteristic curve; CI, confidence interval.

### 3.3 Models validation

Among the 20 included studies, nine performed internal validation only, three reported both internal and external validation, and one study conducted an independent validation of an existing risk score, whereas seven studies did not report any validation following model development.

### 3.4 Results of quality assessment

**Figure 2:**
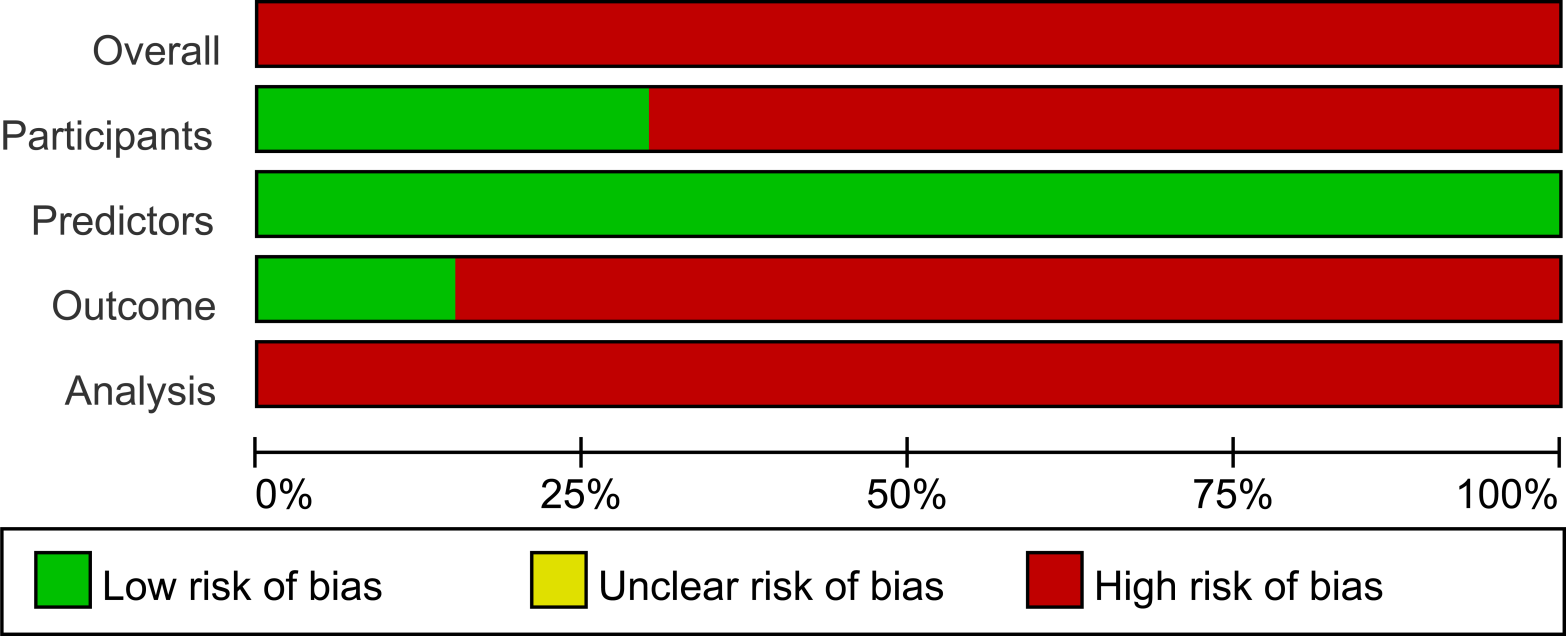
Risk of bias summary: judgements for each domain across included studies (percentage of studies).

**Table 3** summarizes the risk of bias and applicability assessments of the included studies, and **Figure 2** presents the risk-of-bias summary across domains. A thorough evaluation of the studies revealed a high risk of bias, suggesting the presence of methodological challenges in the development or validation processes.

**Table 3:** PROBAST results of the included studies.

Author/year	ROB				Applicability			Overall	
	Participants	Predictors	Outcome	Analysis	Participants	Predictors	Outcome	ROB	Applicability
Cai et al. (2023) (21)	–	+	–	–	–	+	+	–	–
Li Y. et al. (2021) (22)	–	+	–	–	+	+	+	–	+
Lin et al. (2025) (32)	+	+	–	–	–	+	+	–	–
Qin et al. (2023) (23)	–	+	–	–	+	+	+	–	+
Tang et al. (2025) (24)	–	+	–	–	–	+	+	–	–
Chen C. et al. (2025) (33)	+	+	–	–	–	+	+	–	–
Li J.et al. (2024) (34)	+	+	–	–	–	+	+	–	–
Li P.et al. (2024) (25)	–	+	–	–	–	+	+	–	–
Liu H.et al. (2024) (26)	–	+	–	–	–	+	+	–	–
Liu Y.et al. (2024) (27)	–	+	–	–	+	+	+	–	+
Zhang et al. (2022) (28)	–	+	–	–	+	+	+	–	+
Ding et al. (2023) (35)	+	+	+	–	–	+	+	–	–
Chen Z. et al. (2025) (17)	–	+	–	–	+	+	+	–	+
Hao et al. (2025) (18)	–	+	–	–	–	+	+	–	–
Li J.et al. (2023) (36)	+	+	+	–	+	+	+	–	+
Ke et al. (2022) (19)	+	+	+	–	+	+	+	–	+
Hachey et al. (2016) (20)	–	+	–	–	+	+	+	–	+
Peng et al. (2025) (29)	–	+	–	–	+	+	+	–	+
Jin et al. (2025) (30)	–	+	–	–	+	+	+	–	+
Yang et al. (2022) (31)	–	+	–	–	–	+	+	–	–

ROBAST, Prediction model Risk Of Bias Assessment Tool; ROB, risk of bias.

+ indicates low ROB/low concern regarding applicability; - indicates high ROB/high concern regarding application; ? indicates unclear ROB/unclear concern regarding applicability.

In the domain of participants, fourteen studies were adjudged to be at elevated risk of bias, primarily due to the use of inappropriate or insufficiently described data sources, such as single-center retrospective cohorts, unclear participant recruitment procedures, or eligibility criteria that failed to ensure adequate representativeness of the target population (17, 18, 20–31). In the predictor domain, the majority of studies demonstrated adequate selection, measurement, and statistical management of predictors, with no discernible sources of bias identified. Conversely, within the outcomes domain, seventeen studies were classified as high risk of bias, primarily due to the lack of blindness to predictor information during outcome assessment. This potential oversight may have introduced information or assessment bias, thereby compromising the credibility and generalizability of the prediction models (17, 18, 20–34).

Assessment using PROBAST showed that all included studies were rated as high risk of bias in the analysis domain. The recurrent methodological concerns were related to inadequate event numbers relative to model complexity, handling of continuous predictors and missing data, predictor selection procedures, completeness of performance evaluation, and assessment of overfitting or model optimism (11). Sixteen studies had inadequate sample sizes because they did not meet the recommended threshold of more than 20 events per variable (EPV ≥ 20), which may have resulted in unstable coefficient estimates and spurious predictor effects (18, 21, 22, 24–36). Four studies dichotomized or categorized continuous predictors, which may have reduced predictive information and increased the risk of model distortion (25, 27, 29, 32). Eighteen studies handled missing data inadequately. Most relied on complete-case analysis, an approach that may introduce biased estimates (18–21, 23–36). Nineteen studies used univariable screening for predictor selection, a data-driven approach that may inflate optimism and compromise model stability (17–19, 21–36). Thirteen studies did not address issues of overfitting, underfitting, or model optimism. These studies either lacked internal validation or relied solely on apparent performance (18, 19, 22, 23, 25–30, 32, 34, 35). Additionally, 15 studies failed to report information on data complexity, thereby limiting reproducibility and methodological transparency (17, 20–31, 34, 35). 

With respect to applicability risk, ten studies were classified as high risk, and ten studies were classified as low risk. In the participants domain, ten studies were considered to have high applicability risk because the enrolled population was restricted to specific subgroups of lung cancer patients. In contrast, both the predictors and outcomes domains were judged as low applicability risk, as the studies employed commonly used clinical variables and standardized diagnostic criteria for VTE.

### 3.5 Meta-analysis of validation models included in the review

**Figure 3:**
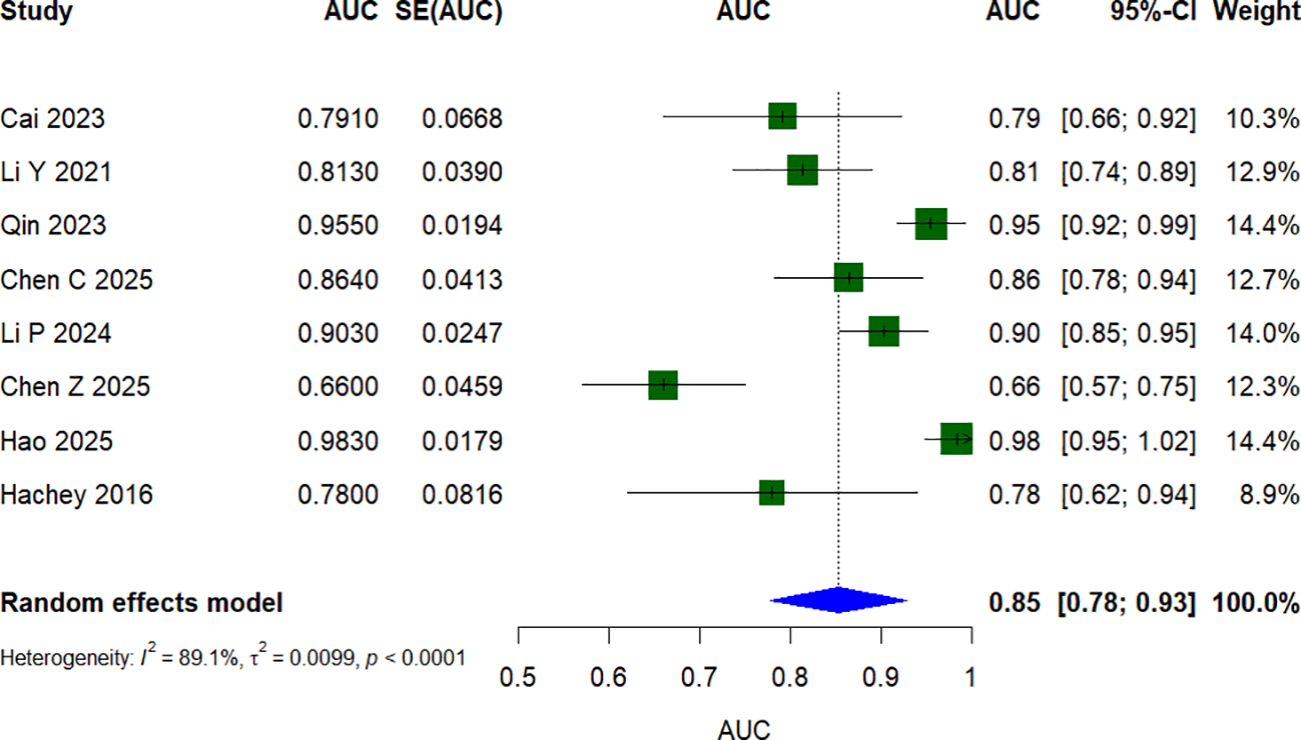
Forest plot of the random effects meta-analysis of pooled AUC estimates for 8 validation models.

**Figure 4:**
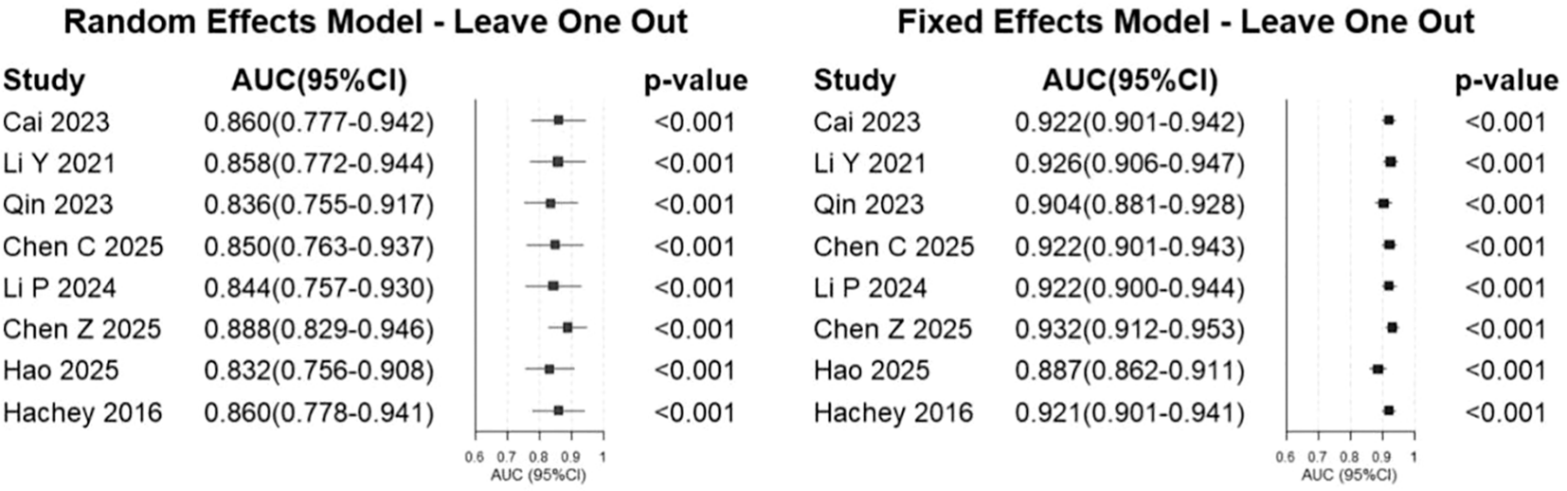
Sensitivity analysis using the leave-one-out model.

Of the 20 identified studies, only eight contributed validation-set AUC estimates that were sufficiently complete and methodologically suitable for quantitative synthesis. Studies without post-development validation, or without extractable and comparable validation performance measures, were not included in the meta-analysis. Among the eight studies included in the quantitative synthesis, AUCs from post-development validation were pooled: external validation AUCs were used when available (Chen C. et al. and Li P. et al.), otherwise internal validation AUCs were used (Cai et al., Li Y. et al., Qin et al., Chen Z. et al., and Hao et al.); for Hachey et al., the AUC was derived from an independent validation of an existing risk score. In the absence of confidence intervals, the Hanley and McNeil method was employed to calculate the standard error from the AUC value and sample size (12, 13). Using a random-effects model, the pooled AUC was 0.85 (95% confidence interval [CI]: 0.78–0.93) (**Figure 3**). The *I*^2^ value was 89.1% (*p* < 0.001), indicating a high degree of heterogeneity among the studies. Leave-one-out sensitivity analysis (**Figure 4**) showed that the pooled results remained relatively stable after sequential exclusion of each study.

## 4 Discussion

Postoperative VTE is a recognized complication of lung cancer surgery with the potential to be prevented (37). Despite the advancements in perioperative management and thromboprophylaxis that have been made over the past decade, VTE continues to be a significant cause of postoperative morbidity and mortality in this population (38). An accurate assessment of VTE risk is therefore crucial to enable early identification and timely preventive strategies, which may substantially improve clinical outcomes. This review discloses an expanding array of risk prediction models that have been developed for postoperative VTE in patients with lung cancer. However, the majority of existing models are derived from single-center datasets in China. Although several models showed moderate to good discrimination in internal or external validation settings, all included studies were judged to have a high risk of bias according to PROBAST, limiting their readiness for routine clinical use. The reported AUC values ranged from 0.66 to 0.979. However, according to the PROBAST checklist, all included studies were judged to have a high risk of bias, thereby limiting the practical utility of these prediction models in real-world clinical practice. The pooled AUC value of the eight models that were validated and included in the meta-analysis was 0.85 (95% CI: 0.78–0.93). Nevertheless, substantial heterogeneity was present across studies, which may be attributed to differences in patient characteristics, surgical procedures, predictor definitions, and methodological approaches. Although the *I*² values were relatively high, the estimated AUCs for all eight validation models exceeded 0.65, and the forest plots showed a generally consistent trend in discriminatory performance. Therefore, this heterogeneity primarily reflects differences in the magnitude of AUC values across studies, rather than conflicting conclusions regarding the models’ discriminatory ability. We therefore emphasize that this heterogeneity should be acknowledged when interpreting the pooled AUC; however, it is unlikely to alter the overall conclusions of this review. Furthermore, several studies incompletely reported key details of model development and validation, which reduced reporting transparency and made critical appraisal more difficult. Future studies should improve reporting in line with the TRIPOD statement and prioritize adequate sample sizes, rigorous methodological design, multicenter external validation, and transparent reporting of model development and validation (39).

Valuable methodological insights can be drawn from the development processes of the included models. For instance, Cai et al. developed their model using a relatively large postoperative lung cancer cohort; however, internal validation was limited to a simple split-sample approach. Split-sample validation is frequently regarded as a form of internal validation. However, it is inadequate for addressing model optimism and may not adequately control for overfitting (39). In contrast, the studies by Chen Z. et al. and Hao et al. also involved substantial datasets but adopted retrospective designs, which increased the risk of bias arising from participant selection, predictor assessment, and outcome determination. Nevertheless, these two studies had methodological strengths in the analysis domain. Chen et al., for instance, employed multiple imputation to address missing data and provided comprehensive assessments of model calibration and discrimination—elements that were often neglected in other studies. It is worth noting that Chen et al. and Hao et al. combined machine learning methods—such as XGBoost and ensemble models—with traditional logistic regression models. This research approach is consistent with previous findings: some issues related to sample size, the handling of continuous variables, and the selection of predictors can be addressed by incorporating machine learning methods into the model-building process (40). However, there is currently a lack of suitable visualization tools for machine learning models in clinical settings. Furthermore, according to the ProBAST assessment, all included research models were found to have a high risk of overall bias; however, among the 20 studies in this review, the models developed by Lin et al., Chen C. et al., and Li P. et al. are of greater clinical value—as they employed more comprehensive validation and calibration processes than most other included models (25, 32, 33). However, models in this study that were not validated after development, or for which validation and calibration reports were incomplete, are not suitable for current clinical use (17–24, 27–31, 34–36). Nevertheless, this review remains valuable for its exploratory nature: it systematically identifies the major methodological flaws and applicability issues of existing models, laying the groundwork for future model optimization, external validation, and clinical translation.

The reported existing prediction models in this review also have certain clinical implications. D-dimer-related indicators and age were the most frequently incorporated predictors, appearing in 15 and nine models, respectively. This pattern suggests that coagulation activation and patient-related vulnerability are consistently considered relevant to postoperative VTE risk. D-dimer is a well-recognized marker of fibrin formation and degradation, but differences in measurement timing and cutoff values across studies limit comparability and may reduce model transportability (41, 42). Age may reflect reduced physiological reserve, comorbidity burden, and postoperative immobility, which are clinically plausible contributors to thrombotic risk (32). Caprini-related scores and operation time or surgical duration were also commonly incorporated, each appearing in seven models. Operation time may capture surgical complexity, prolonged anesthesia exposure, tissue injury, and impaired venous return, but inconsistent definitions and cutoffs across studies may reduce the comparability and stability of its predictive role (38). Preoperative chemotherapy, reported in four models, may reflect treatment-related endothelial injury and cancer-associated hypercoagulability, suggesting that both surgery-related and treatment-related factors should be considered when refining postoperative VTE prediction models (43). Although Caprini-based risk assessment tools have been explored in patients undergoing lung cancer surgery, their specificity appears limited in some studies. In particular, Ding et al. reported that the modified Caprini risk assessment model had a specificity of only 38.9%, suggesting that this model may overestimate postoperative VTE risk in some patients (35). Taken together, these findings suggest that postoperative VTE risk in patients with lung cancer is shaped by both general clinical factors and surgery-related characteristics; however, inconsistencies in predictor definitions and the limited specificity of general tools indicate the need for more refined models tailored to this population.

## 5 Limitations

The present review has several limitations. First, the majority of the studies were conducted in China, which may limit the generalizability of the study’s findings to a broader international population. The applicability of these prediction models is influenced by various factors, including regional management strategies, blood clot prevention strategies, and patient characteristics. Therefore, future studies should focus on testing these models with a more diverse group of lung cancer patients and developing VTE risk models. Second, because of the heterogeneity in the clinical features and methods used in the included studies, a significant discrepancy was observed in the meta-analysis. This discrepancy may be related to differences in population characteristics, VTE definitions, follow-up duration, and study methods. Given that only eight models with validated, complete, and comparable AUC estimates were included, it is not possible to further investigate the heterogeneity using either sub-group analysis or meta-regression. In the future, studies should employ standardized outcome definitions and transparent reporting methods to conduct more rigorous external validation. It is important to note that this review includes only English and Chinese studies, which may introduce language bias, resulting in the omission of key findings in other languages. 

## 6 Conclusion

This systematic review, which encompassed twenty studies that evaluated twenty postoperative VTE prediction models for lung cancer patients, revealed that the pooled AUC of the eight models that were externally or internally validated was 0.85 [95% confidence interval (CI): 0.78–0.93], suggesting that these models possess a satisfactory overall discriminatory capacity. However, an evaluation of the included studies using the PROBAST tool revealed a high risk of bias. Additionally, several studies indicated concerns regarding the applicability of the findings across various clinical settings. The existing prediction models do not meet PROBAST standards, underscoring the necessity for methodological refinement prior to their reliable implementation in clinical practice. In order to enhance the quality and clinical relevance of future research endeavors, investigators are strongly encouraged to adhere to the PROBAST framework and to adhere to the reporting recommendations outlined in the TRIPOD statement. Future efforts should prioritize the development of robust models supported by larger sample sizes, rigorous study designs, standardized predictor handling, and multicenter external validation to enhance generalizability and facilitate clinical translation.

## Data Availability

The original contributions presented in the study are included in the article/**Supplementary Material**. Further inquiries can be directed to the corresponding authors.
